# Supplementary material for: Effect of Dietary Rumen-Protected L-Tryptophan Supplementation on Growth Performance, Blood Hematological and Biochemical Profiles, and Gene Expression in Korean Native Steers under Cold Environment
Source: Animals (Basel). 2019 Nov 27;9(12):1036. doi: 10.3390/ani9121036 (PMC6941001; doi:10.3390/ani9121036)
Supplement: Supplementary file 1 [file animals-09-01036-s001.pdf]

Table S1. Primer sequences used for quantitative reverse transcription PCR assay

Gene symbol	Gene name	Annealing temperature, °C	Primers
C/EBPα	CCAAT/enhancer-binding protein alpha	60.3	F: CCGTGGACAAGAACAGCAACGA<br>R: GGCGGTCATTGTCACTGGTCAG
FABP4	Fatty acid binding protein 4	60.0	F: GTGTGATGCATTTGTAGGT<br>R: CTGGTGGCAGTGACACCAT
PPARγ	Peroxisome proliferator-activated receptor gamma	61.0	F: TGGAGACCGCCCAGGTTTGC<br>R: AGCTGGGAGGACTCGGGGTG
MyoD	Myoblast determination protein	59.6	F: AGAGTTGCTTTGCCAGAG<br>R: CTGCCTGCCGTATAAACA
MYF6	Myogenic factor 6	60.7	F: GAAGGAGGGACAAGCATTGA<br>R: GAGGAAATGCTGTCCACGAT
DESMIN	Desmin	61.0	F: GGACCTGCTCAATGTCAAGA<br>R: GGAAGTTGAGGGCAGAGAAG
18S	18S ribosomal RNA	51.0	F: ACCCATTCGAACGTCTGCCCTATT<br>R: TCCTTGGATTGTGGTAGCCGTTTCT
GAPDH	Glyceraldehyde-3-phosphate dehydrogenase	60.0	F: GGCAAGGTCATCCCTGAG<br>R: GCAGGTCAGATCCACAACAG
RPLP0	Ribosomal protein lateral stalk subunit P0	55.0	F: CAACCCTGAAGTGCTTGACAT<br>R: AGGCAGATGGATCAGCCA
